# Anlotinib-containing regimen for advanced small-cell lung cancer: A protocol of meta-analysis

**DOI:** 10.1371/journal.pone.0247494

**Published:** 2021-03-11

**Authors:** Guocan Yu, Qingshan Cai, Xudong Xu, Yanqin Shen, Kan Xu

**Affiliations:** Zhejiang Tuberculosis Diagnosis and Treatment Center, Affiliated Hangzhou Chest Hospital, Zhejiang University School of Medicine, Hangzhou, Zhejiang, China; Mansoura University, EGYPT

## Abstract

**Background:**

Small cell lung cancer (SCLC) is a highly malignant lung cancer with a very poor prognosis. Clinical treatment options for SCLC are still limited, especially for patients who have failed first or second line therapy. Anlotinib is a potentially beneficial new treatment option for SCLC. The aim of this meta-analysis is to evaluate the efficacy and safety of anlotinib-containing regimen for the treatment of SCLC.

**Methods:**

We will search SinoMed, Wanfang Database, China National Knowledge Infrastructure, Embase, Cochrane Library, and PubMed for relevant articles that may meet the criteria published before March 31, 2021. We will perform a meta-analysis to evaluate the efficacy and safety of anlotinib-containing regimen for the treatment of SCLC. Clinical randomized controlled trials comparing anlotinib-containing regimens with other treatment regimens for advanced SCLC will be included in this study. The risk of bias will be evaluated for each included study using the Cochrane Handbook for Systematic Reviews of Interventions. We will use RevMan 5.3 software for statistical analysis of the data.

**Results:**

The results of this study will provide evidence of anlotinib-containing regimens for advanced SCLC, and provide clinicians and patients with another convenient and effective treatment regimen for SCLC. This meta-analysis will be submitted to a peer-reviewed journal for publication.

**Conclusion:**

This meta-analysis will provide clinical evidence of anlotinib-containing regimens for advanced SCLC, which may or may not be found for anlotinib use.

**Systematic review registration:**

INPLASY202110034.

## Introduction

Lung cancer is one of the most common and deadliest tumors in the world [1]. Lung cancer can be divided into two categories: non-small cell lung cancer (NSCLC) and small cell lung cancer (SCLC). NSCLC accounts for about 85% of all lung cancers and SCLC accounts for about 15% [2, 3]. Although the proportion of SCLC is small, its prognosis is worse than that of NSCLC. SCLC progresses rapidly and is prone to metastasis elsewhere, such as lymph nodes, bone, and brain, even at the time of diagnosis [4]. Platinum-containing chemotherapy is the first-line option for SCLC treatment. Although most SCLC responds well to first-line chemotherapy, a majority of SCLC relapses and progresses within 6 months [5]. Some studies have shown that SCLC can benefit from immunotherapy [6, 7], but its application is still limited by its high price and some of its serious toxic side effects. Current treatment modalities are limited when progression after chemotherapy and/or immunotherapy [8]. These characteristics of SCLC make its 5-year survival rate quite low, only about 2% [9].

Neovascularization plays an important role in the growth, proliferation and metastasis of solid tumors. Anti-angiogenic drugs can inhibit tumor neovascularization, degrade existing tumor blood vessels and reduce tumor blood supply, thus inhibiting tumor growth. Anti-angiogenic therapy is emerging as an effective anti-tumor treatment in addition to chemotherapy, targeted therapy and immunotherapy, which is also applicable to lung cancer [10, 11]. Anlotinib, an oral novel small-molecule multitarget tyrosine kinase inhibitor (TKI), which could inhibit tumor angiogenesis and proliferation [12]. By inhibiting tumor blood supply, anlotinib has shown promising therapeutic effects in a variety of tumors, such as endometrial, ovarian, and cervical cancers [13]. Anlotinib has also shown very good therapeutic efficacy in NSCLC [14] and benefits in SCLC [15], but there is a lack of systematic evidence for the use of anlotinib in SCLC. The purpose of this meta-analysis is to further evaluate the efficacy and toxicity of anlotinib-containing regimens in the treatment of advanced SCLC.

## Methods

Following this study protocol, we will conduct a meta-analysis to assess the efficacy and safety of anlotinib-containing regimen for advanced SCLC. The preferred reporting items for systematic review and meta-analysis protocols (PRISMA-P) statement is followed to perform this protocol [16]. We will report the final results of this meta-analysis based on the Preferred Reporting Items for Systematic Reviews and Meta-Analyses (PRISMA) statements [17]. On the International Platform of Registered Systematic Review and Meta-analysis Protocols (INPLASY), the protocol has been registered with the registration number of INPLASY202110034 [18].

### Information sources

We will search SinoMed, Wanfang Database, China National Knowledge Infrastructure (CNKI), Embase, Cochrane Library, and PubMed for relevant articles that may meet the criteria published before March 31, 2021. We will perform an up to date search, where the most recent publication date cut off is within the last 12 months. We will also evaluate whether the references cited in the reviews meet the criteria to find additional articles.

### Search strategy

The two authors (Guocan Yu and Qingshan Cai) will collaborate to design relevant search strategies for different databases and conduct searches in the databases to identify relevant articles. We do not place any restrictions on language or dates. The search strategy for PubMed is showed as follows:

#1 "Small Cell Lung Carcinoma"[Mesh] OR “Small Cell Lung Cancer” OR “Oat Cell Lung Cancer” OR “Small Cell Cancer of The Lung” OR “Carcinoma, Small Cell Lung” OR “Oat Cell Carcinoma of Lung”
#2 "anlotinib" [Supplementary Concept] OR AL3818
#3 #1 AND #2

The SinoMed, Wanfang Database, CNKI, Embase, and Cochrane Library search strategies will be similar to Pubmed.

### Eligibility criteria

#### Types of studies

Clinical randomized controlled trials (RCTs) comparing anlotinib-containing regimens with other treatment regimens for advanced SCLC will be included in this study. Retrospective studies, single arm studies, articles published in languages other than Chinese or English, conference reports, studies with only abstracts reported but no full texts, and case reports will be excluded.

#### Types of participants

Participants with histopathologically or cytologically confirmed advanced SCLC and treated with anlotinib-containing regimen or other regimens. We do not apply any restrictions in terms of age, gender, and ethnicity.

#### Types of interventions

Anlotinib-containing regimen as an intervention in the observation group. Regimen without anlotinib (including placebo) as a control group.

#### Outcomes

The primary outcomes of this study will be progression-free survival (PFS) and overall survival (OS). The secondary outcomes will be the objective response rate (ORR), disease control rate (DCR), and adverse events (AEs).

*Study selection*. The candidate articles obtained from the search will be imported into Endnote X9.2 for management. After removing duplicate articles through Endnote, two authors will independently investigate the title, abstract and then full texts of each article to determine whether the article meet the inclusion criteria designed in this protocol. When there are disagreements between the two authors, it will be resolved by discussing with a third author (Kan Xu).

*Data extraction*. The same two authors in the study screening phase will independently extract the relevant data from the included articles. They will cross-check to find inconsistent data and discuss with a third author to resolve the disagreement.

We will extract the following data from the included articles: country, name of first author, publication year, study design, number of participants, participants characteristics (such as age, sex, smoking history, and brain metastasis), treatment regimen, number of lines of therapy, and median PFS, OS with hazard ratios (HRs) and their 95% confidence intervals (CIs), ORR, DCR, and AEs.

*Risk of bias*. The same two authors will independently assess the risk of bias of each included article. The risk of bias will be evaluated for each included study using the Cochrane Handbook for Systematic Reviews of Interventions [19]. We will assess the risk of bias according to the following ranges: selection bias, performance bias, detection bias, attrition bias, reporting bias, and other biases. If more than 10 articles are included, then we will use funnel plots for publication bias assessment [20]. If publication bias exists, we will use the fill and trim method to further analyze publication bias in the studies.

*Evidence evaluation*. We will evaluate all the strength of the body of evidence based on The Grading of Recommendations Assessment, Development and Evaluation (GRADE) guideline [21]. The quality of evidence will be classified into 4 levels: high, moderate, low, and very low.

*Statistical analysis*. We will use RevMan 5.3 software (Copenhagen: The Nordic Cochrane Centre, The Cochrane Collaboration, 2014) for statistical analysis of the data. The Engauge Digitizer 4.1 software will be used to extract survival data from the Kaplan-Meier curves if only Kaplan-Meier curves are shown in the study and median PFS and/or OS with HRs are not directly reported. We will calculate pooled HRs for PFS and OS, risk ratios (RRs) for ORRs and DCRs, and odds ratios (ORs) for different AEs. Q-statistic will be used to evaluate the statistical heterogeneity between studies [22]. Heterogeneity between studies will be considered statistically significant when the *P*-value of the Q-statistic is less than 0.1 or an I^2^ is greater than 50% [23]. Data will be analyzed using a fixed-effects model when heterogeneity between studies is insignificant and a random-effects model when heterogeneity between studies was significant. Subgroup analyses will be performed on various parameters, such as age, sex, smoking history, number of lines of therapy, brain metastasis, and number of metastases to reduce heterogeneity. By removing one study and reporting the analysis results with and without this study to perform a sensitivity analysis to determine whether the study is a high-risk study. A *P* value less than 0.05 was considered statistically different. A pooled HR greater than 1 indicated a greater rate of progression or death following treatment with anlotinib-containing regimen, a pooled RR greater than 1 indicated a greater overall response, and a pooled OR greater than 1 indicated greater toxicity of treatment with anlotinib-containing regimen.

## Discussion

SCLC is a highly malignant lung cancer with a very poor prognosis. To improve the prognosis of SCLC, effective treatment options are needed, but current clinical treatment options are still limited, especially for patients who have failed first or second line therapy. The application of anlotinib as a potentially beneficial new treatment option for SCLC is also very convenient. To our knowledge, this will be the first meta-analysis to evaluate the efficacy and safety of anlotinib-containing regimen for the treatment of SCLC. We hope that this study will provide clinicians and patients with another convenient and effective treatment regimen for SCLC, thus improving the prognosis of SCLC.

## Supporting information

S1 Checklist PRISMA-P 2015 checklist: Recommended items to address in a systematic review protocol. (DOC)
